# Multicenter study correlating molecular characteristics and clinical outcomes of cancer cases with patient-derived organoids

**DOI:** 10.1186/s13046-025-03437-0

**Published:** 2025-07-02

**Authors:** Paloma Navarro, Tatiana P. Grazioso, Arantzazu Barquín, Maria Barba, Mónica Yagüe, Carlos Millán, Irene López, Elena Sevillano, Miguel Quiralte, Paloma Fernández, Diego Losada, Eduardo Caleiras, Julia Calzas, Beatriz Jiménez, Sergio Ruiz-Llorente, Juan Justo, Félix Guerrero, Vital Hevia, Raquel Martín, Francisco José Pérez-Rodriguez, Julia Tejerina, Mario Prieto, Paula Comune, Juan Francisco Rodriguez-Moreno, Jesús García-Donás

**Affiliations:** 1 Laboratory of Innovation in Oncology, Genitourinary, Gynecological and Skin Cancer Unit, HM Clara Campal Comprehensive Cancer Centre (CIOCC) MADRID. Sanchinarro HM University Hospital, HM Hospitals, Madrid, Spain; 2 HM Faculty of Health Sciences, Camilo José Cela University, Madrid, Spain; 3 HM Hospitals Health Research Institute, Madrid, Spain; 4 Institute of Applied Molecular Medicine (IMMA), Department of Basic Medical Sciences, Facultad de Medicina, Universidad San Pablo CEU, CEU Universities, Urbanización Montepríncipe, Madrid, Spain; 5 Gynecological Department, HM University Hospital, Madrid, Sanchinarro Spain; 6 Histopathology Core Unit, Spanish National Cancer Center (CNIO), Madrid, Spain; 7 Lung Cancer Unit, HM Clara Campal Comprehensive Cancer Centre (CIOCC) Madrid, Sanchinarro HM University Hospital, HM Hospitals, Madrid, Spain; 8 Medical Oncology Department, Fuenlabrada University Hospital, Madrid, Spain; 9 Departamento de Biomedicina y Biotecnología, Área de Genética, Universidad de Alcalá, Madrid, Spain; 10 Roc Clinic, Madrid, Spain; 11 Department of Urology, Prostate Cancer Division, HM Hospitales, Madrid, Spain; 12 Department of Urology, HM Hospitales, Kidney Division, Madrid, Spain; 13 Department of Pathology, Therapeutic Target Laboratory, Hospital Universitario HM Sanchinarro, Madrid, Spain; 14 Hospital Universitario San Jorge, Huesca, Spain; 15 Vivía Biotech, Madrid, Spain

**Keywords:** Precision Medicine, Patient-derived organoids, Drug screening

## Abstract

**Background:**

3D-spatial interaction between cancer cells influences tumor behavior, making it essential to replicate tumor structures for predicting patient outcomes.

**Methods:**

We collected data from three multicenter prospective studies to evaluate the ability to establish Patient-Derived Organoids (PDOs) from different biological samples and timepoints, correlating their characteristics and drug sensitivity with clinical outcomes.

**Results:**

From 184 patients (17 tumor types), 249 samples were collected: 149 (59.8%) from tumor tissue, 61 (24.5%) from peritoneal fluids, 39 (15.7%) from peripheral blood. Success rates for PDO establishment were 39.5%, 34.4%, and 25.6%, respectively. PDOs reproduced pathological and immunohistochemical patterns of source tumors, with pathogenic variants confirmed in 84% (21/25). In a series of 13 baseline and sequential PDOs from 9 patients undergoing treatment, responses to therapy mirrored patient responses during therapy.

**Conclusions:**

PDOs preserve tumor features, reflect disease progression, and predict treatment responses, providing valuable models to complement molecular testing in precision medicine.

**Supplementary Information:**

The online version contains supplementary material available at 10.1186/s13046-025-03437-0.

## Background

Cancer is the leading cause of death in the world. According to the World Health Organization (WHO), about 10 million people died from this entity in 2020, and about 19 million people were newly diagnosed [1].

Despite significant advances in the management of this disease, only a minority of patients benefit from genetic testing and precision medicine today [2, 3]. On one hand, most medications already approved for cancer have overlapping clinical indications, while on the other hand, many mechanisms of resistance are driven by adaptations in the epigenetic profiles of tumors or their microenvironment, which cannot be properly assessed by current molecular assays [4–7]. Consequently, determining the best therapeutic option for each case remains a difficult decision that both physicians and patients must face daily.

Additionally, the identification of driver mutations leading to the overactivation of downstream pathways has reached a plateau. As a result, new therapeutic strategies, such as targeting the tumor microenvironment, enhancing the immune response, or inhibiting transcription factors, have been widely adopted in daily practice [6]. Since traditional 2D cell cultures cannot capture this complexity, they are insufficient for supporting the rational development of new compounds, testing combinations of existing drugs, or predicting patient responses to therapies [8].

Finally, new phenomena, such as the bystander effect of antibody–drug conjugates (ADCs), which have demonstrated clear therapeutic impact, can only be properly studied using 3D structures that replicate the spatial distribution of cancer cells [9]. Therefore, comprehensive models capable of mimicking in vitro the spatial organization of tumors and their behavior are essential for both developing new therapeutic approaches and matching each case with the most appropriate treatment option [10].

Genetically engineered animal models and patient-derived tumor xenografts (PDTX) in immunocompromised or humanized mice have been proposed to more closely resemble the complexity of tumors and their physiological conditions [11–13]. However, the microenvironment is invariably replaced by murine cells, which do not behave like the original tumor ecosystem. Additionally, these models are expensive, time-consuming, and raise concerns about animal suffering within the scientific community and society at large [13].

In recent decades, 3D culture technologies have enabled the development of novel human tissue and disease models [14, 15]. Cancer patient-derived organoids (PDOs) are three-dimensional cultures that attempt to retain the genetic heterogeneity and closely mimic the morphological characteristics of the original tumors. These organoids are generated by isolating cancer cells from a patient's biopsy or surgical sample and growing them in a matrix within specific culture media [16]. Thus, PDOs could serve not only to study cancer biology and test potential therapies but also to personalize patient treatment [17–21].

Unfortunately, to date, most studies have focused on determining the in vitro characteristics of organoids without analyzing their correlation with the real outcomes of the original patients. Furthermore, few research groups have experience establishing organoids from alternative sources, such as peripheral blood or peritoneal fluids, and even then, only with a limited number of samples [22–26]. This could be key when trying to implement this technology in daily practice, where obtaining tumor tissue through surgical biopsies or percutaneous punctures is a major challenge. Finally, there is no experience assessing how PDOs evolve throughout the disease or comparing PDOs from paired samples of a given patient.

We aimed to establish PDOs from samples of different origins and at different time points from cancer patients. We compared their anatomo-pathological characteristics and genetic background with those of the original tumors and assessed whether their in vitro drug sensitivity mirrored the actual outcomes of the source cases throughout the disease, in a multicentric environment. Only this kind of study, directly comparing in vitro results with real-life outcomes, can help incorporate new and better models into drug discovery and precision medicine.

## Methods

### Ethics

Data was obtained through three observational studies focused on identifying biomarkers in cancer patients that were performed in accordance with the ethical principles of the Declaration of Helsinki and was consistent with ICH/Good Clinical Practice. The studies protocols were approved by the central ethics committee at our institution. All patients provided written consent before sample collection. All samples were collected along standard procedures indicated as daily practice. The obtention of enough tissue or biological fluids for diagnostic procedures was always prioritized thus, investigational samples were only collected from remnant material.

All studies were observational thus, no randomization or blinding procedure was implemented. Also, since the main objective is purely descriptive, no formal power analysis was required.

### Clinical data collection

Clinical data and radiological images were extracted from the original medical reports after anonymization.

### Sample collection

All samples were obtained from remnants during intervention indicated as part of daily practice (tumor or metastasis resection or gross needle biopsy for tumor tissue, ascites drainage for tumor cytology or peripheral blood extraction for circulating tumoral cells (CTCs). Diagnostic procedures were always prioritized. Samples were included in Phosphate-Buffered Saline (PBS) 1 × and maintained at 4ºC until processing, in all cases before 24 h after collection.

### Tissue dissociation and organoid establishment

Tissue fragments of approximately 500–1000 mm3 were obtained immediately after tumor resection in the operating room. Non-necrotic lesions with solid or papillary growth were collected and processed. Samples were maintained at 4ºC in PBS 1 × until processing, not later than 24 h after collection. Tissue fragments were cut into 2–3 mm pieces, washed with cold PBS for several rounds and then dissociated into small clusters or single cells by digestion with Type IV Collagenase (Life Technologies; #17,101,015) 1 mg/mL and DNAse 0.5 mg/ml during 30–40 min at 37ºC, with vortex each 10 min. The homogenate was then filtered through a 70um filter (ClearLine #141379C) and then through a 40um filter (ClearLine #141378C). Filter was washed with Basal Medium (Advanced DMEM F12 + 1% Pen-Strep + Glutamax 1% + Hepes 1%). The cell suspension were then spun at 1000 rpm to create a cell pellet that was treated with 5 ml of ACK Lysing buffer for 5–10 min to eliminate erytrocytes and then washed once with basal culture media.

Cancerous ascites from peritoneal fluid were centrifuged at 4 °C for 10 min at 3000 rpm. The cell pellet was then washed with PBS 1 × and passed through 70 and 40um filters and processed as done with the digested homogenates from solid tumors.

For general culture, once a pellet of tumor cells was obtained, the cells were mixed with growth factor–reduced Matrigel (Corning; catalog number CB-40230C), with the final concentration of Matrigel at 75% and approximately 10,000 or more cells/cell groups per 10 μL droplet of Matrigel. The suspension was then rapidly plated into a 24-well plate with 20 μL of suspension per well. Once the Matrigel was solidified, 250 μL of general culture medium was added to each well. The general culture medium was specific for each histology (see Table S6). The number of wells to seed tumor cells was empirically determined depending on the amount of cell pellets recovered.

### Organoid culture

The organoid culture medium was changed every 3 days. In general, the passage was conducted at 70–80% confluence. For that, the medium was aspirated, and organoids were collected with cold PBS 1X into a 15 ml tube. After centrifugation at 1200 rpm, 1 ml of TrypLE Express (Gibco#12,605–010) was added to the cell pellet and incubated for 5 min at 37ºC. Cells were then split 1:2–1:3 depending on the growth speed.

Organoids from 3–4 70% of confluence wells were criopreserved in Recovery™ Cell Culture Freezing Medium (Fisher #12648010).

### Immunohistochemistry characterization

For Immunohistochemistry characterization, organoids after fixation with formalin 10%, were paraffin-embedded. Tissue samples were cut at 3 µm, mounted on superfrost®plus slides and dried overnight. For immunohistochemistry, an automated immunostaining platform Autostainer Link, Dako, was used. Antigen retrieval was performed with High pH buffer, Dako, Agilent; endogenous peroxidase was blocked (peroxide hydrogen at 3%) and slides were then incubated with the appropriate primary antibody as detailed: Mouse Monoclonal, anti- P53 (DO-7, Ready to use, Agilent Cat# IR616, RRID:AB_3669092); Mouse Monoclonal, anti- WT1 (6F-H2, Ready to use, Agilent Cat# IR055, RRID:AB_3669093) and Rabbit polyclonal, anti- Pax8 (1/400, Proteintech Cat# 10,336–1-AP, RRID:AB_2236705). After the primary antibodies, the slides were incubated with the corresponding secondary antibodies (P53 and WT1 with anti-mouse and Pax8 with anti-rabbit) and EnVision FLEX +, Dako's horseradish peroxidase-conjugated visualization systems (Agilent Cat# SM802, RRID:AB_3075507). The immunohistochemical reaction was revealed with 3, 30-diaminobenzidine tetrahydrochloride (DAB) included in the Dako Flex kit.

Finally, nuclei were counterstained with Carazzi's haematoxylin, slides were dehydrated, rinsed and mounted with permanent mounting medium for microscopic evaluation. Positive control sections known to be primary antibody positive were included for each staining run.

### Co-culture of renal PDOs and autologous Tumor-Infiltrating lymphocytes (TILs)

#### Tumor-infiltrating lymphocytes isolation and amplification

After surgically obtaining renal tumor a portion of the tumor sample was used for organoid generation following the protocol described in section 2, while another portion were utilized to isolate tumor-infiltrating lymphocytes (TILs). We based on the protocol previously published by Rosenberg laboratory [27]. Briefly, small pieces of tumor (usually 6) measuring about 1 to 2 mm in each dimension were cut with a sharp scalpel from different areas around the tumor specimen. A single tumor fragment was placed in each well of a 24-well tissue culture plate with 1 mL of complete medium (CM) plus 6000 IU per mL of rhIL-2 (Peprotech, US). CM consisted of RPMI 1640, 25 mmol/L HEPES pH 7.2, 100 U/mL penicillin, 100 μg/mL streptomycin, 2 mmol/L-glutamine, supplemented with 10% human serum. The plates were placed in a humidified 37 °C incubator with 5% CO2 and cultured until lymphocyte growth was evident. Each fragment was inspected about every other day using a low-power inverted microscope to monitor the extrusion and proliferation of lymphocytes. Whether or not lymphocyte growth was visible, half of the medium was replaced in all wells no later than 1 week after culture initiation. Typically, about 1 to 2 weeks after culture initiation, a dense lymphocytic carpet would cover a portion of the plate surrounding each fragment. When any well became almost confluent, the contents were mixed vigorously, split into two daughter wells and filled to 2 mL per well with CM plus 6000 IU/mL IL-2. Subsequently, the cultures were split to maintain a cell density of 0.8–1.6 × 106 cells/mL, or half of the media was replaced at least twice weekly.

#### TILs characterization

For TILs characterization, they were stained with two different panels of antibodies: Panel 1 included anti-CD326 (Epcam)-APC (Miltenyi Biotec Cat# 130–113–260, RRID:AB_2726061) to identify epithelial cells and anti-CD45-PerCP (Miltenyi Biotec Cat# 130–094–975, RRID:AB_10831670) to identify hematological cells; and Panel 2 included anti-CD3-FITC clone HIT3a (BioLegend Cat# 300,306, RRID:AB_314042) to identify T lymphocytes, anti-CD4-PE/Cyanine7 clone OKT4 (BioLegend Cat# 317,413, RRID:AB_571958) to identify Helper T Lymphocytes and anti-CD8-BV605 clone SK1 (BioLegend Cat# 344,741, RRID:AB_2566512) to identify Cytotoxic T Lymphocytes. After 15 min of staining, cells were washed and resuspended in PBS 0.2%BSA 3 mM EDTA and analyzed in 1. Attune™ NxT Cytometer (Thermo Fisher). Data analysis was performed with FlowJo software (RRID:SCR_008520).

#### Co-culture renal PDOs-autologous TILs

Two days before co-culture, PDOs were dissociated with TrypLE (Gibco), counted, resuspended in 75% Matrigel in basal medium and seeded in 3ul-drops in 96-well blacked-wall plates (Greiner #655,986), with 3000 cells/drop. After two days of culture, the organoid’s medium was removed and a suspension of TILs was added to the corresponding wells in two different proportions: 10:1 or 20: 1 (TILs:tumoral cells), in the presence or absence of Ipilimumab, an anti-CTLA4 antibody used as immunotherapy. The co-culture was maintained for five days.

#### Co-culture viability measurement by fluorescence

After five days of co-culture in the presence or absence of Ipilimumab, the PDOs cell death was quantify following the fluorometric method previously described by Bode et al. [28]. Briefly, the medium was removed and the PDOs were stained with propidium iodide (PI) and Hoechst at a final concentration of 10 μg/ml each. Staining solution (dyes in PBS). Organoids were stained for 30 min at 37 °C, 5% CO2 for subsequent analysis on the plate reader. Then, the staining medium was removed and replenished with fresh phenol-red free medium before analysis. Stained organoids still embedded in Matrigel were measured in a plate reader (Varioskan, ThermoFisher). Excitation and emission wavelengths for PI were 535 m and 617 nm, respectively, and for Hoechst 361 and 486 nm, respectively. During the measurement, all wells were first measured for PI fluorescence and after a 30-s delay for Hoechst fluorescence. The PI/Hoechst ratio was calculated by dividing PI by Hoechst RFUs. Using PI/Hoechst ratio, treatment specific organoid cell death was calculated: each sample was divided by the mean of all staurosporine (STS)-treated organoids and resulting values multiplied by 100. Then, mean of all untreated (ut) organoids was subtracted to set ut organoids to 0.

### Genomic characterization by Sanger sequencing

Organoids were harvested from matrigel and genomic DNA isolated using the Nucleospin Gel And Pcr Clean-Up (Machinery-Nagel #677,497) following the instructions of the manufacturer.

Specific primers (Table S5) were designed to amplify the targeted gene regions by PCR using organoid genomic DNA and the Supreme NZYTaq II 2 × Green Master Mix (Nzytech RRID:SCR_016772,#MB360). The amplicon was verified using gel electrophoresis, purified with the E.Z.N.A Gel Extraction Kit (Omega Bio-Tek #D2500-02) according to the manufacturer’s protocol, and Sanger-sequenced by Macrogene Spain (Madrid, Spain).

### Drug screening

Organoids were harvested from matrigel and dissociated in single-cell with Triplex. The cell suspension was resuspended in 75% Matrigel in basal medium and seeded in 3ul-drops in 96-well blacked-wall plates (Greiner #655,986). The tumor-specific culture medium was added, and organoids were allowed to grow during 1–2 weeks. Different dilutions of drugs (0.25xCmax, 0.5xCmax, Cmax, 2xCmax, and 4xCmax) (Table S5) [29–32] were used in triplicates using as control the treatment with vehicle (DMSO 0.3%). After 72 h treatment, viability was measured with the CellTiter-Glo® 2.0 Cell Viability Assay, in a Varioskan (Thermo). The sensitivity score was calculated as the inverse of the AUC (1/AUC), for each drug/PDO with GraphPad Prism (RRID:SCR_002798).

### CTC isolation and culture

Blood samples were processed within 30 min of collection from patients, following the protocol described in Xiao et al. [33] with small modifications. Briefly, Samples were mixed with 1 × PBS 2% FBS at 1:1 volume/volume ratio at room temperature and poured into a 50 mL tube Septmate containing 15 ml of Lymphoprep. Samples were spun for at 1200xg for 10 min at 4◦C, at which point plasma, buffy coat, Lymphoprep, and RBCs formed four distinct layers.

The plasma and buffy coat layers were combined, harvested, and mixed with 1 × PBS 2%FBS to 50 mL final volume and spun at 300 × g for 10 min at 25◦C. Steps taken after this were done under sterile conditions.

Once spun, the supernatant was aspirated and two additional washes using 1 × PBS were performed. Washes were spun at 300 × g for 10 min at 4◦C.

Upon completion of the final wash, cells were resuspended in culture medium and plated for short-term cultures at 37◦C.

Cultures were supplemented with fresh medium every 3 days and washed every 6 days with 1 × PBS by centrifugation of the supernatant at 100 × g for 4 min at 4◦C.

### Immunoprofiling

Once received at the laboratory, the tumor mass was measured, weighed, and processed for matrix digestion and cell disaggregation. Then, the tumor piece was placed in a culture cell dish with a small volume of tumor growth media for mechanical digestion using scalpels. Tumor fragments were transferred to a tissue dissociation tube (Tube C, Miltenyi) and a cocktail of collagenases and DNase enzymes were added to promote matrix digestion. The mix was incubated at 37ºC for 30 min followed by automatic processing at gentleMACS™ Dissociator equipment (Miltenyi). The digested sample was filtered (100 and 40 µm sterile filters), and residual erythrocytes were lysed by incubation with lysis buffer for 5 to 10 min at 4ºC (ELB, Qiagen). The final cell pellet was resuspended in tumor growth media and an aliquot was used for flow cytometry characterization.

An aliquot of patient tumor cells was incubated with different antibodies cocktails to perform the sample baseline phenotyping by flow cytometry. In all samples, the cell viability marker Live Dead (APC-Cy7 conjugated, Invitrogen) was used to discriminate alive and dead cells, anti-EpCAM (PE conjugated, clone EBA-1, BD Biosciences Cat# 347,198, RRID:AB_400262) and anti-PanCK (PE-Cy7 conjugated, clone C-11, Life Technologies) conjugated antibodies were used to identify epithelial-derived tumor cells. Anti-CD45 (PO conjugated, clone HI30, Thermo Fisher Scientific Cat# MHCD4530, RRID:AB_10376143) was used to identify hematological cell populations and anti-CD5 (PECy7 conjugated, clone UCHT2, BioLegend Cat# 300,622, RRID:AB_2275812) was used to identify infiltrated T lymphocytes (TILs). In some specific samples also count on anti-EGFR (PE clone AY13,, BioLegend Cat# 352,903, RRID:AB_10898161, or FITC conjugated, clone AY13, Biolegend) and anti-E-Cadherin (PECy7 conjugated, clone 67A4, BioLegend Cat# 324,116, RRID:AB_2563096) as additional tumor markers and on additional flow cytometry panels, designed to check TILs phenotype including, per example, anti-PD1 (FITC conjugated, clone EH12.247, BioLegend Cat# 379,205, RRID:AB_2922605), anti-CCR7 (PE-Cy7 conjugated, clone G043H7, BioLegend Cat# 353,225, RRID:AB_11125576), anti-CD45RA (APC conjugated, clone HI100, BD Biosciences Cat# 550,855, RRID:AB_398468), anti-CD25 (APC-Cy7 conjugated, clone M-A251, BD Biosciences Cat# 557,753, RRID:AB_396859), anti-CD4 (PO conjugated, clone OKT4, Biolegend), anti-CD8 (PECy7 conjugated, clone SK1, BioLegend Cat# 980,910, RRID:AB_2876774 or CD8A, APC conjugated, clone HIT8a, BioLegend Cat# 300,911, RRID:AB_314115) and anti-FOXP3 (BV conjugated, clone 206D, BioLegend Cat# 320,123, RRID:AB_2561338).

The staining was analyzed at Omnicyt™ flow cytometer combined with Autosamplers CytKick Max. Data analysis was performed using FlowJo™ software (RRID:SCR_008520).

### Statistics

Categorical data were summarized in tables presenting frequencies and percentages. Continuous data were summarized using the mean, median, standard deviation and range. The number of non-evaluable outcomes and of missing data were also provided.

The statistical evaluation was performed using the software GraphPad Prism (RRID:SCR_002798). First, the Shapiro–Wilk normality test was performed to assess the distribution of the data. For samples that met the normality criteria, a parametric t-test was applied to evaluate differences between groups. In cases where the data did not follow a normal distribution, the non-parametric Mann–Whitney U test was used for analysis. These tests ensure statistical validity by considering the nature of the data.

Since this is a descriptive study, no formal calculation of sample size was performed.

## Results

### Establishment of PDOs

**Fig. 1:**
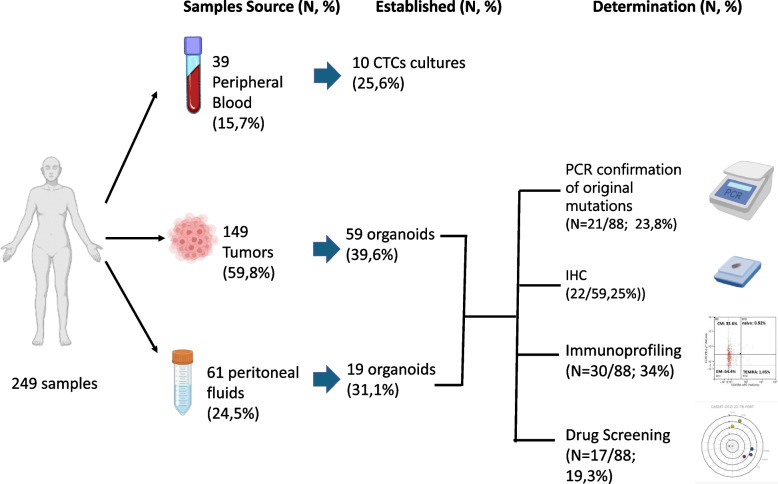
Flow chart of samples collected for PDO establishment and characterization. Samples included: peripheral blood (obtained through venipuncture), tumor (obtained through biopsy or percutaneous punction) and peritoneal fluids (peritoneal washings along a surgical procedure or malignant ascites). The success rate of organoid establishment is depicted, alongside the analyses conducted: immunohistochemistry (IHC), determination by PCR of the presence of DNA pathogenic variants identified in the source tumor (PCR confirmation of original mutation), characterization of the immune cell populations present in the source tumor (Immuneprofiling), study of sensitivity to different drugs to compare in vitro response with the real outcome of patients (Drug screening). Partially created in BioRender.com

Between January 2020 and March 2024, 184 patients who underwent routine blood extraction 39 (21.2%), peritoneal drainage 1 (0.54%), tumor biopsy 37 (20.1%) or oncological surgery 107 (58.1%) at three collaborating institutions were included in the study. 120 were women (65.2%) and 64 men (34.8%), with a median age of 66 years (range [26–91]); 64.3 (range [26–87]) for women and 66 (range [27–91]) for men (Fig. 1, Table 1 and Table S2).

**Table 1:** Patients demographics

Demographics	Number (N)	Percentage (%)
Patients (*N* = 184)		
Women	120	65,2
Men	64	34,8
Age (median-range)		
Total	66 (26–91)	
Women	64,3 (26–87)	
Men	66 (37–91)	
Type of tumor		
Ovary	77	41,8
Prostate	35	19,0
Renal	27	14,7
Endometrium	8	4,3
Lung	6	3,3
Bladder	5	2,7
Colorectal	5	2,7
Melanoma	5	2,7
Uterine sarcoma	5	2,7
Cervix	4	2,2
Merkel	2	1,1
Basocelular	1	0,5
Ovarian Granulosa	1	0,5
Pancreas	1	0,5
Adrenal	1	0,5
Struma ovarii	1	0,5
Stage (TNM)		
I-II	27	14,7
III-IV	124	67,4
UKN	33	17,9
Previous lines of treatment		
None	100	54,3
1	31	16,8
2	21	11,4
≥ 3	31	16,8
UKN	1	0,5
Samples (*N* = 249)		
Source		
Tumors	149	59,8
Peritoneal fluids	61	24,5
Peripheral Blood	39	15,7
Number per patient		
Single	148	59,4
Multiple Synchronous	101	40,6
Multiple metachronous	9	4,9

*UKN* Unknown

Overall, 249 samples were collected (39 [15.7%]) from peripheral blood, 61 [24.5%] from peritoneal washings or malignant ascites and 149 [59.8%] from tumor tissue) (Fig. 1). We collected multiple samples in 105 (42.2%) cases; 101 (40.5%) were “synchronous” and 4 (1.6%) “metachronous” (meaning that were obtained at the same or different timepoints respectively). Metachronous samples were collected from twelve patients (nine who underwent surgery in two occasions, two who underwent surgery three times and one that required peritoneal drainage in four occasions). Paired samples (meaning samples that were obtained from different sources in a same patient) were collected in 50 cases (50 tumor tissue and 51 peritoneal fluids) (Table S2).

Tumors collected presented 17 primary origins: 77 ovary (41.8%), 35 prostate (19%), 27 renal (14.7%), 8 endometrium (4.3%),6 lung (3.3%) and 27 others (15%). Most of the cases (67.4%) were stage III or IV.

By the time of sample collection 100 (54.3%) of patients were treatment naïve while 31 (16.8%), 21 (11.4%), 31 (16.8%) had received one, two or three or more lines of therapy respectively. This data was not available in 1 (0.5%) case.

Once obtained, samples were processed as described in the Material and Methods section. PDOs could be successfully established from 88 of the 249 samples processed, for an overall success rate of 36.1%. (Figure S1, Table S3).

Regarding ovarian cancer (the most frequent tumor in this series) PDO establishment success rate was 46/139 (33%) (3/139[2.1%] for samples from peripheral blood, 21/139 [15.1%] for samples from peritoneal fluids and 22/139[15.8%] for samples from tumor tissue). High grade serous was the most abundant histology and yield a 31/84 [36.9%] success rate.

Regarding paired samples success rates from tumor vs peritoneal wash from the same patient/surgery, were 31.4% (16/51) vs 28% (14/50) respectively. In eight cases (16%) PDO establishment was successful from both the tumor and the peritoneal wash, and in 31 cases (62%), any sample was successful.

### PDOs characterization

#### Immune profiling

**Fig. 2:**
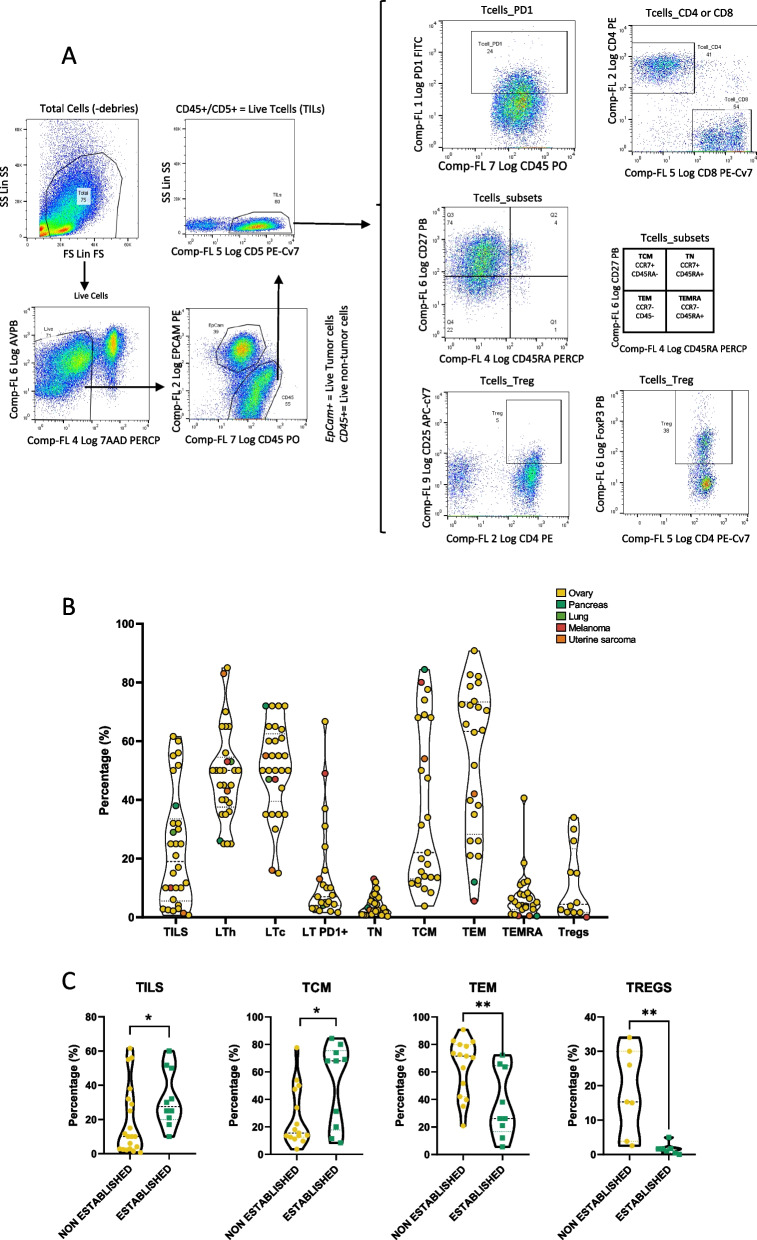
Flow cytometry gating strategy for the study of the immune infiltrate of source tumors (**A**-**B**) and correlation with PDO establishment (**C**). **A** Gating strategy used for immune profiling by flow cytometry, showing the sequential steps to identify and quantify immune cell populations. Two different strategies to detect Tregs cells are shown. **B** Percentage of the different immune populations per patient. The different colors reflect the type of tumor as shown in legend (**C**) Immune populations with significant differences between samples that successfully generated organoids and those that did not. TILs, Tumor Infiltrating lymphocytes; LTh, T Healper lymphocytes; LTc, T cytotoxic lymphocytes; TCM, Central Memory T cells; TEM, Effector Memory T cells; TN, Naïve T cells; TEMRA, Terminal Effector Tcells. *p < 0.05; **p < 0.01

In order to describe the immune microenvironment of source samples, explore associations between immune infiltration and PDO feasibility and determine suitability for further establishment PDOs preserving the original immune landscape (immune organoids described in Sect. 5), we performed a flow cytometry analysis of seven T cells subpopulations (Helper T cells (Th) (CD45 +/CD5 +/CD4 +), Cytotoxic T cells (Tc) (CD45 +/CD5 +/CD8 +), Central Memory T cells (TCM) (CCR7 +/CD45RA-), Effector Memory T Cells (TEM) (CCR7-/CD45RA-), Terminal Effector T cells(TEMRA) (CCR7-/CD45RA +), Naïve T Cells (TN) (CCR7 +/CD45RA +) and Regulatory T Cells (Tregs), in a series of 31 tumor samples (Fig. 2A, B; Table S4). Organoids were successfully established from 10 (32.2% success rate), that showed an enrichment of TILs (27,5% vs 10%, [*p* = 0.042] and TCM (51.5%vs26.5%, [*p* = 0.023]. In contrast TEM and Tregs showed to be enriched in those samples that failed (63.62% vs 36.70% [*P* = 0.009] and 9.4% vs 1,65% [*p* = 0,0045] respectively) (Table S4, Fig. 2C).

#### Immunohistochemical profiling

**Fig. 3:**
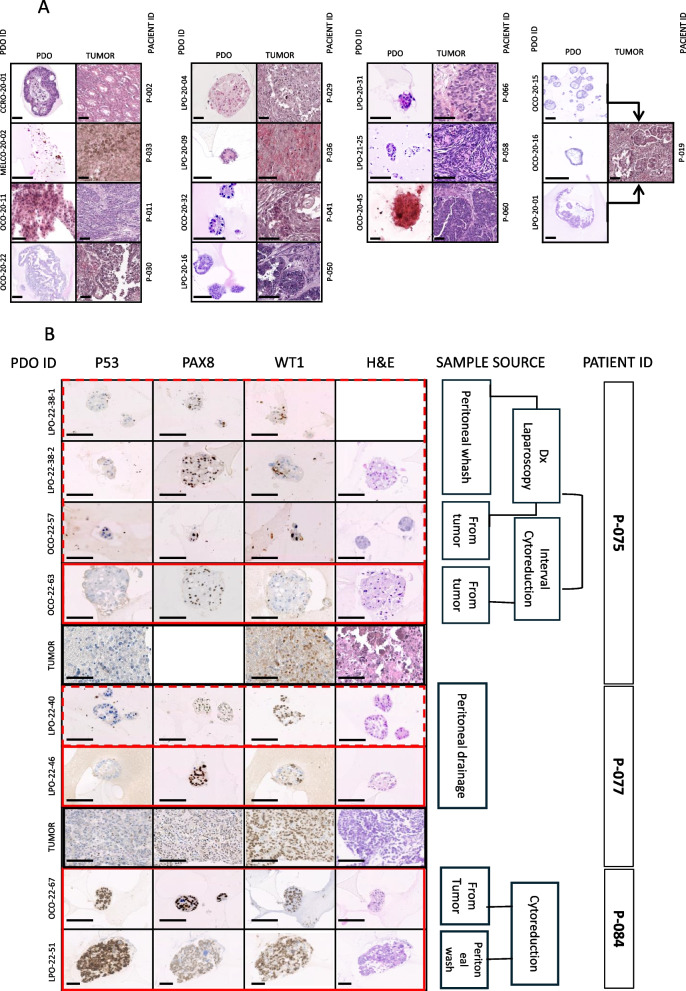
Correlation of morphology and IHC patterns between PDOs and source tumors and between PDOs from paired samples (**A**) H&E staining of paraffin-embedded organoids and their source tumors (**B**) Immunohistochemical staining of ovarian cancer markers in PDOs and source tumors (PAX8, p53, and WT1). Scale bar = 100um

Up to 22 of the 90 established PDOs reached an adequate number and size for immunohistochemical (IHC) profiling (at least 4 confluent wells of a 24-well plate). After fixation and formalin-paraffin embedment hematoxylin–eosin and IHC stainnings for routine diagnostic markers, were performed. Most (20 [91%]) reproduced the histological pattern observed in the corresponding tumors and preserved a similar expression of IHC markers. (Fig. 3 and S2)-(Fig. 3).

#### Genetic profiling

**Fig. 4:**
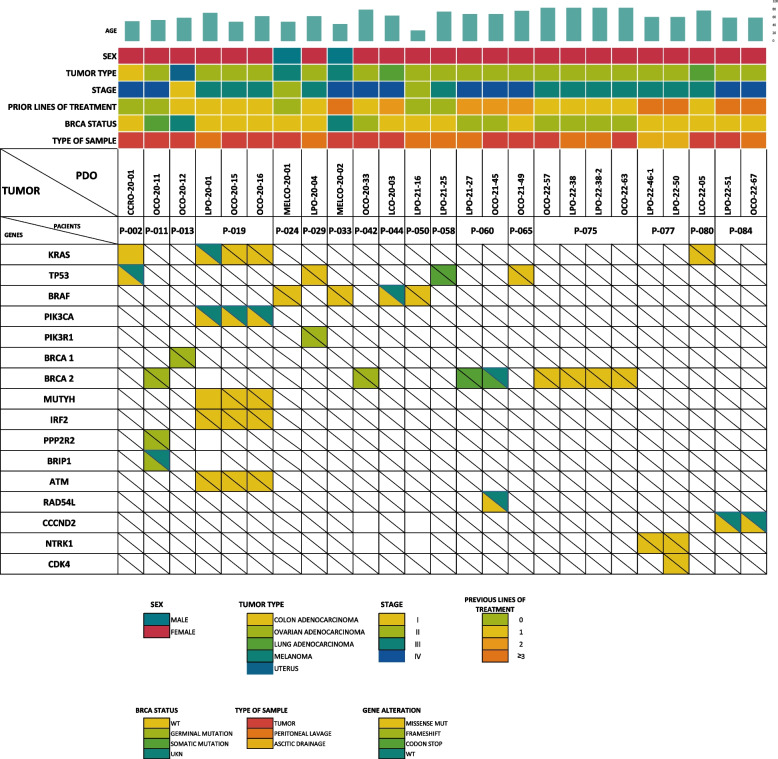
Correlation of the genetic background of PDOs and source tumors The panel displays the presence or absence in PDOs of pathogenic variants originally identified in the source tumors. Also, clinical and pathological data of the patients from whom samples were collected are represented (sex, tumor type, disease stage, number of prior treatment lines, and the type of sample obtained (peripheral blood, tumor or peritoneal fluids)

In order to confirm whether PDOs preserved the genetical background of source tumors we extracted DNA from 27 organoids whose corresponding tumors had been studied through Next Generation Sequencing (NGS) as part of their routine medical practice. PCR sequencing regarding 16 different genes could be performed in 25 (92.6%) PDOs. The presence of at least one original pathogenic variant was confirmed in 21/25 (84%) of the samples (Table 2, Fig. 4 and Table S4).

**Table 2:**
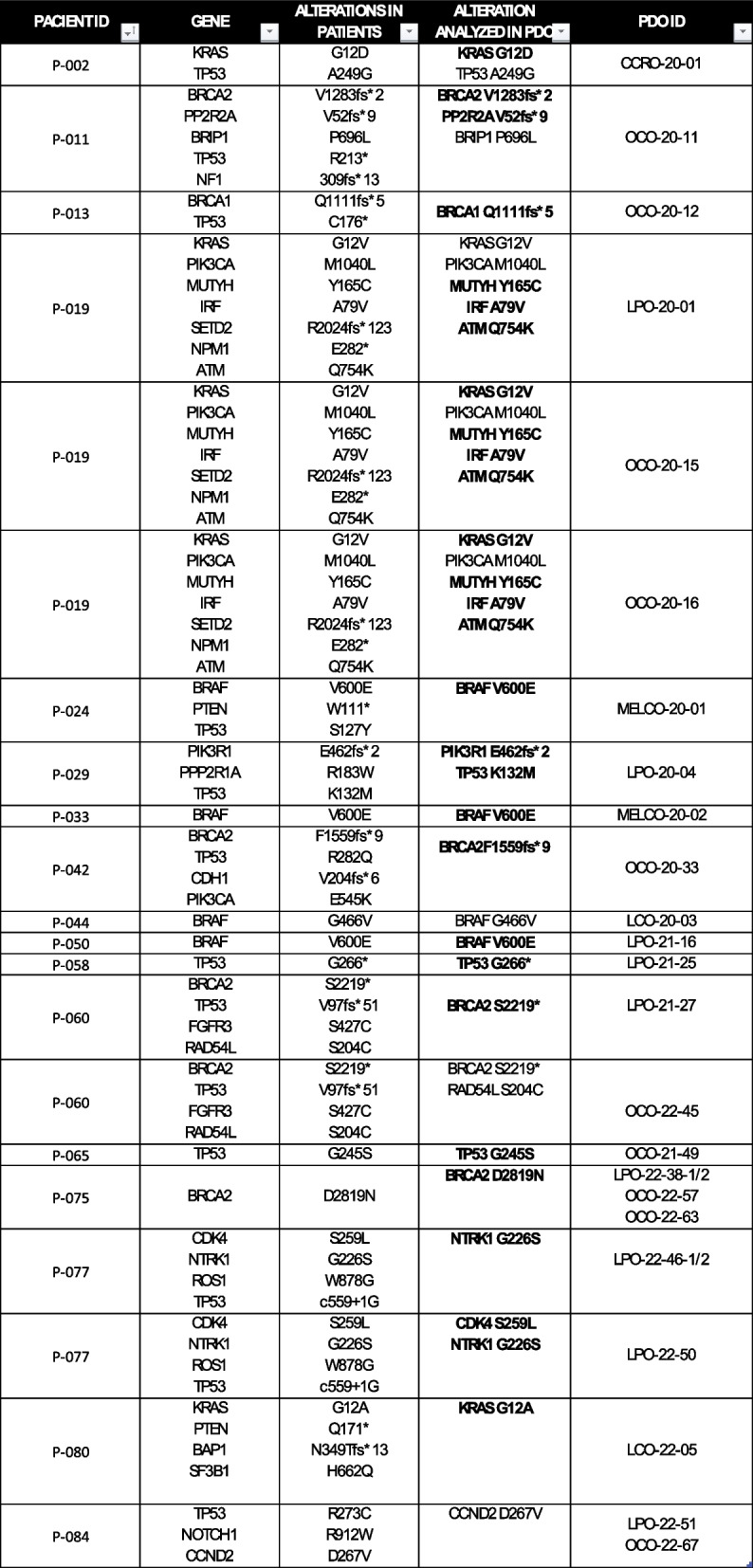
Relation of genes and specific alterations found in tumors

The genes that finally could be detected in PDOs are marked in bold

### Correlation between drug sensitivity of PDOs and real clinical outcome of patients

**Fig. 5:**
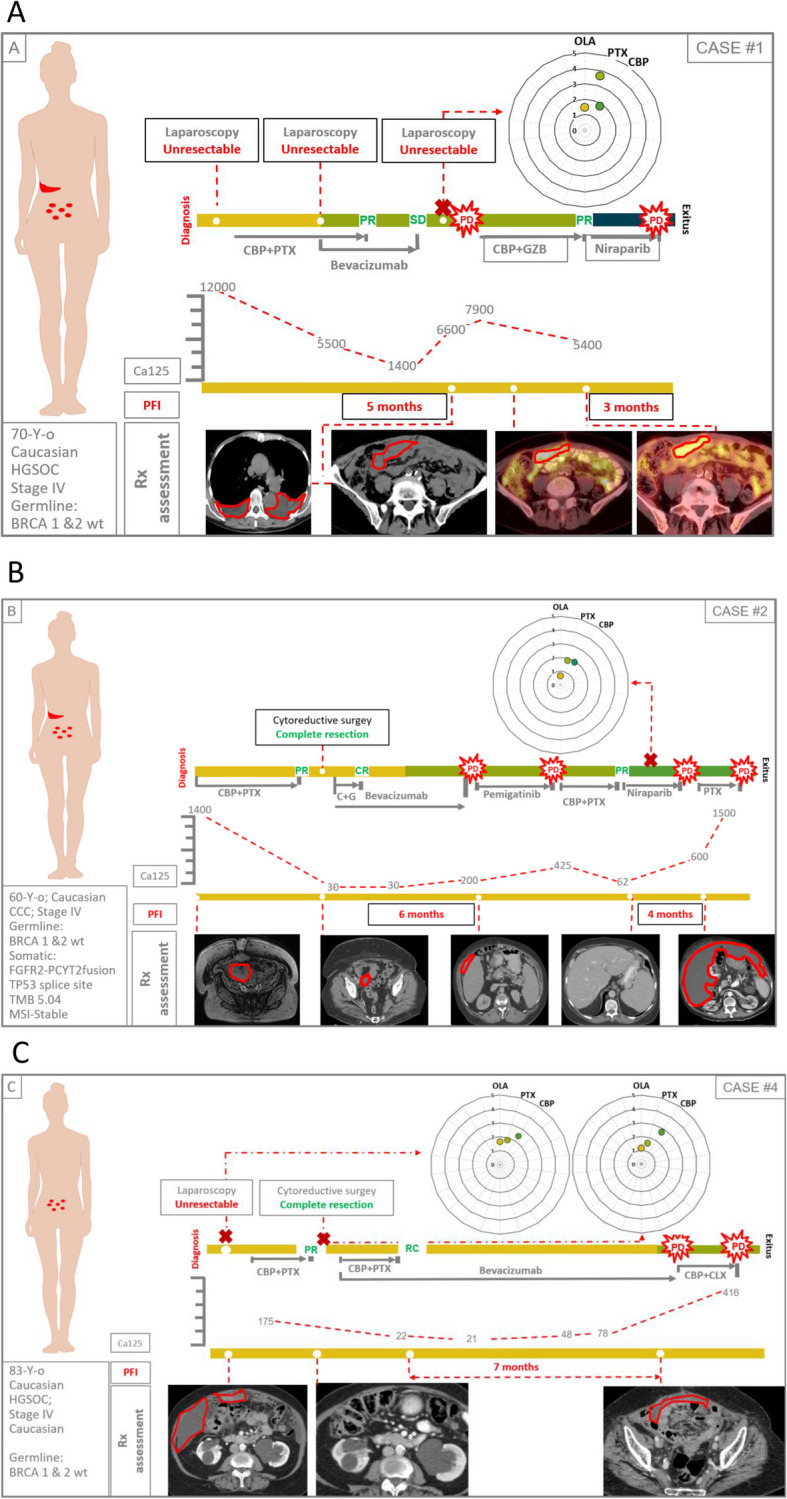
Timelines comparing PDO drug sensitivity with real outcome of source patients (cases #1–3). **A**-**C** Graphical summaries of source patients clinical and molecular characteristics and their clinical evolution and PDO drug sensitivity score

**Fig. 6:**
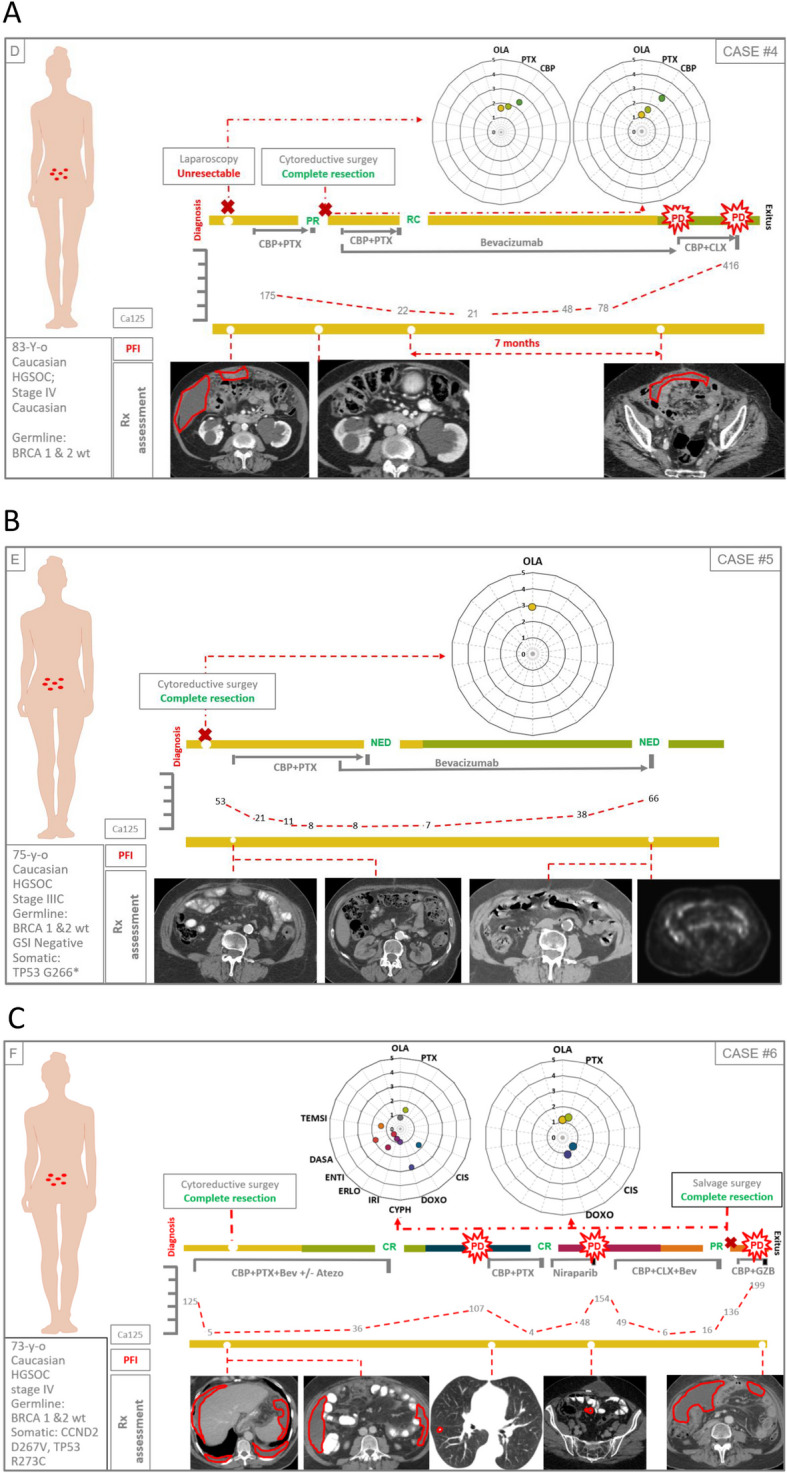
Timelines comparing PDO drug sensitivity with real outcome of source patients (cases #4–6). **A**-**C** Graphical summaries of source patients clinical and molecular characteristics and their clinical evolution and PDO drug sensitivity score

**Fig. 7:**
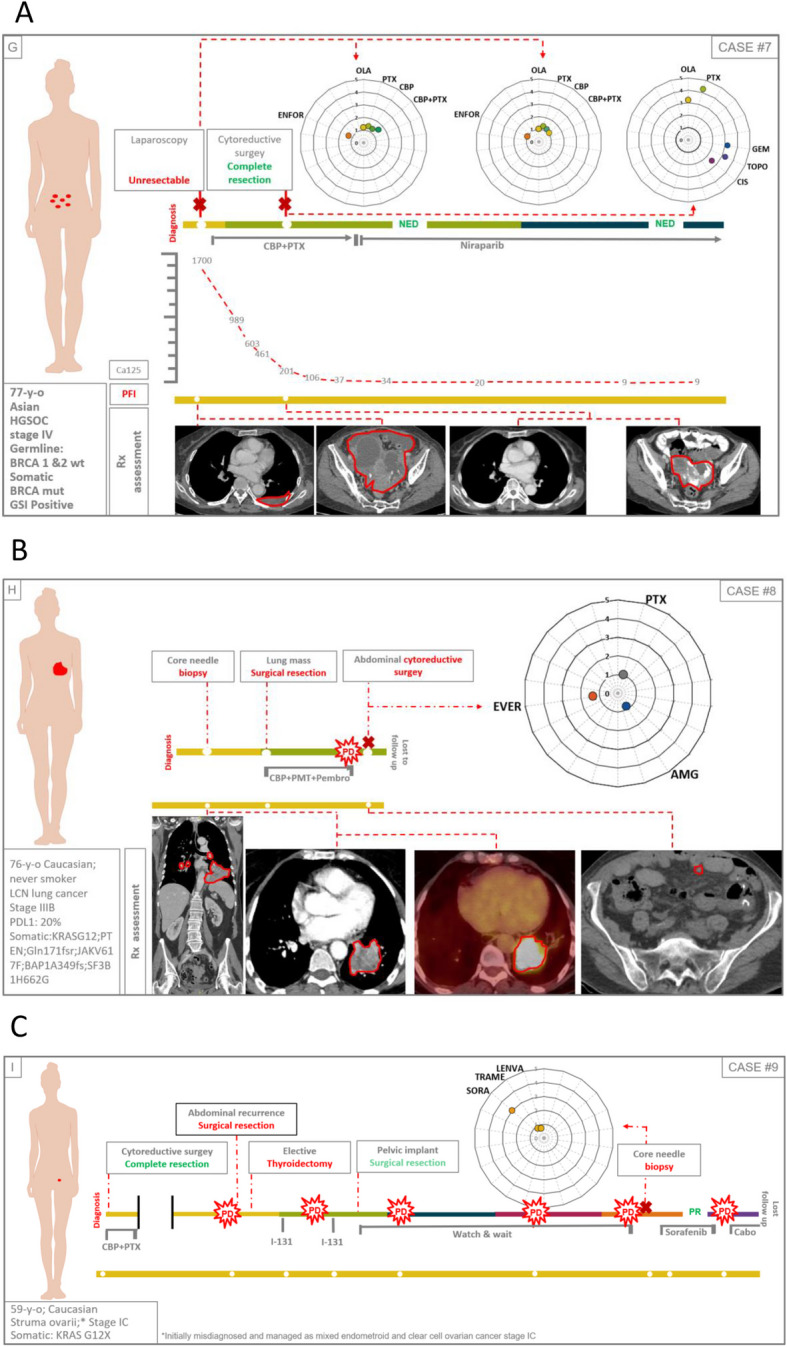
Timelines comparing PDO drug sensitivity with real outcome of source patients (cases #7–9). **A**-**C** Graphical summaries of source patients clinical and molecular characteristics and their clinical evolution and PDO drug sensitivity score

**Fig. 8:**
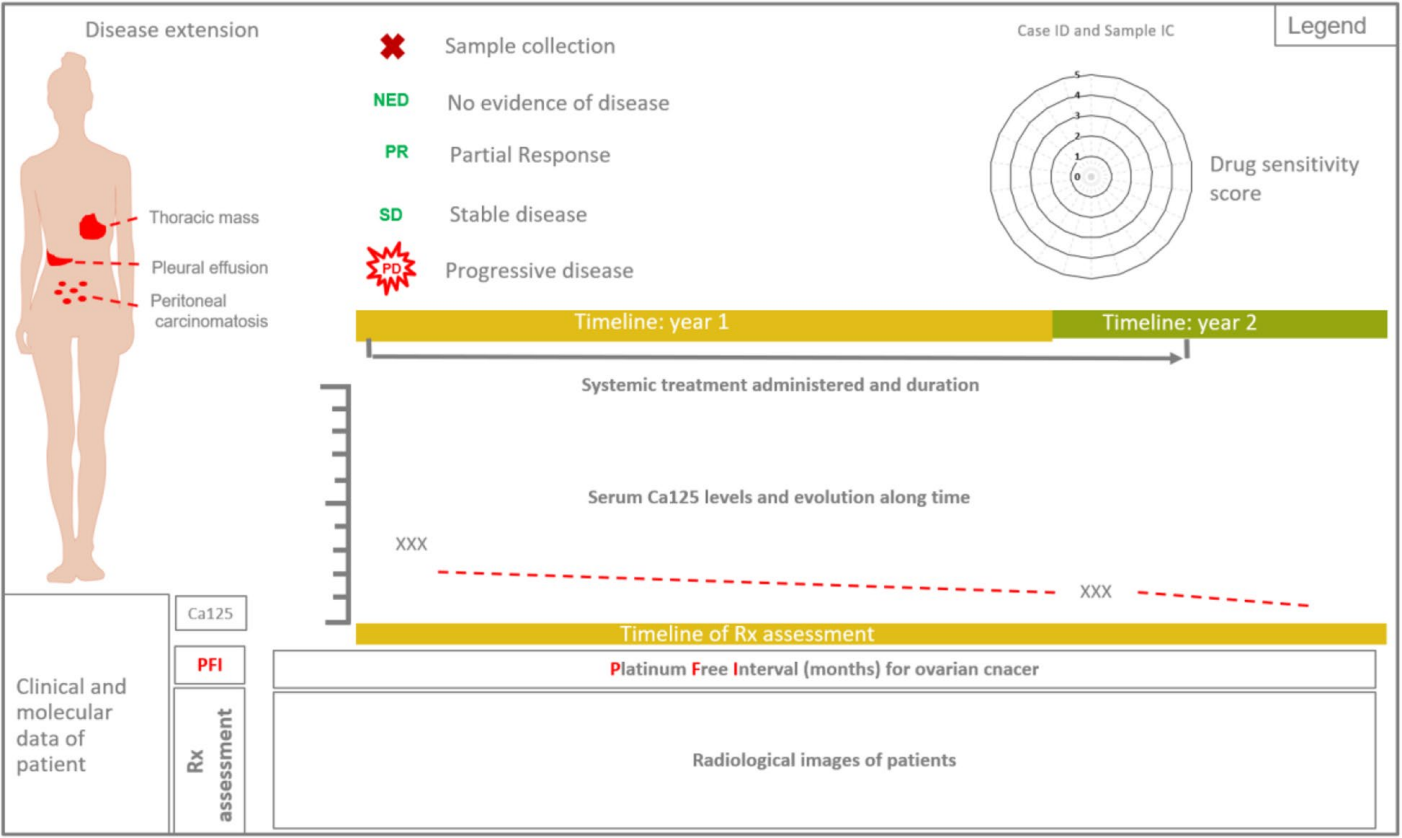
Legend for the correct interpretation of Figs. 5, 6 and 7

**Fig. 9:**
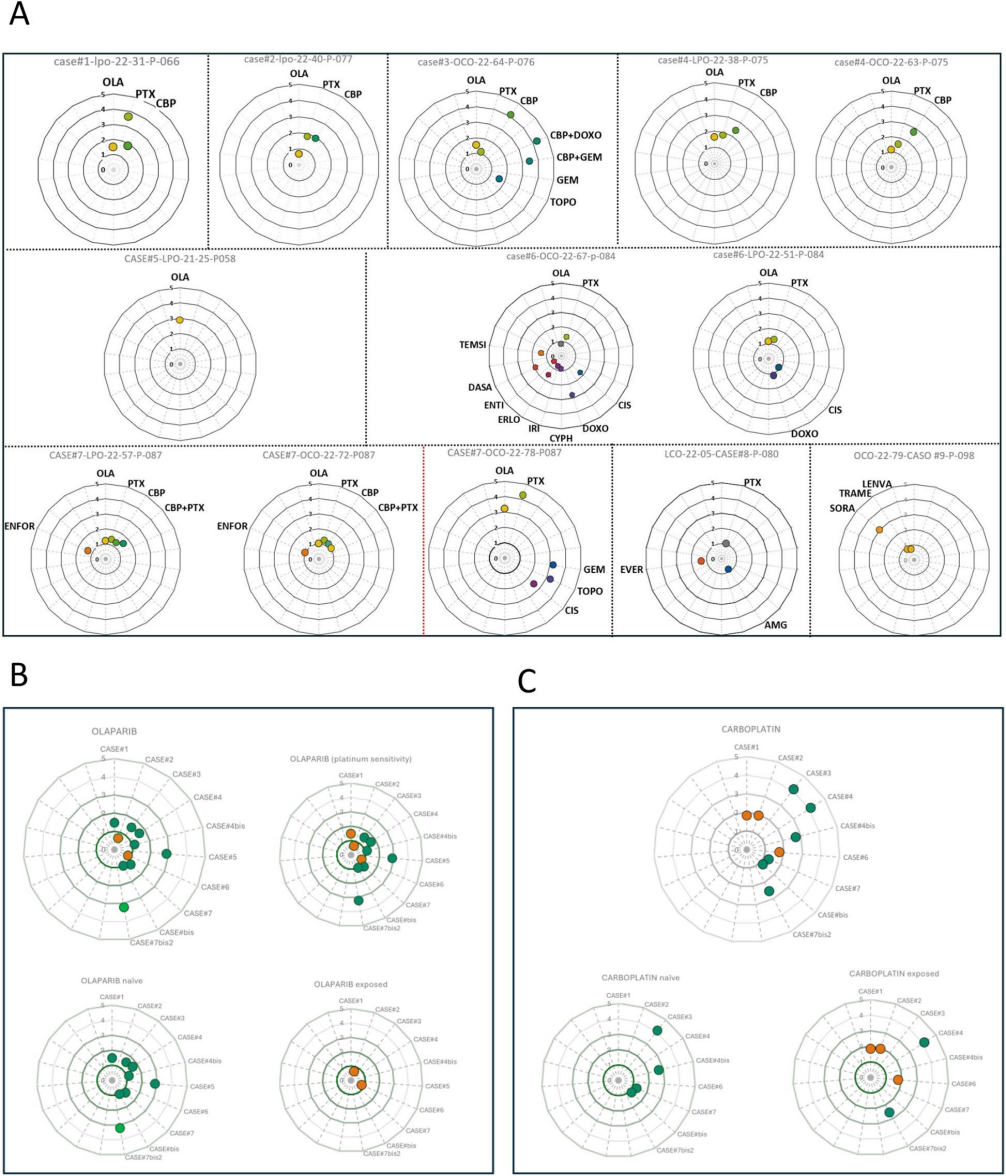
Drug sensitivity scores. **A** Drug sensitivity score of all PDOs (paired synchronous samples are presented within the same square; metachronous samples are separated by a red dot line; PDOs from different patients are separated by black dot lines). **B** and **C** respectively: olaparib and platinum derivates sensitivity scores of PDOs; orange and green circles represent platinum resistant and sensitive patients (respectively); naïve: PDOs established from patients that had not received the drug by the time of sample collection; exposed: PDOs established from patients that had already received the drug by the time of sample collection

We compared the in vitro response of PDOs to therapy with the real clinical outcome of patients in a series of 13 PDOs from nine cases with advanced disease (six high grade serous ovarian cancer, one clear cell ovarian cancer, one large cell neuroendocrine lung cancer and one struma ovarii [an ultra rare histological variant of ovarian cancer that mimics thyroid carcinoma]) (Figs. 5, 6, 7, 8 and 9). At least one PDO from tumor tissue was established in all cases. Paired PDOs were established in four cases: two metachronous (one before and one after neoadjuvant chemotherapy) in cases #4 and #7 and two synchronous (one from tumor tissue and one from peritoneal wash) in cases #6 and #7.

Drugs to be screened were agreed with the attending physicians of the source patients based on the information they considered more relevant in every case: to predict future response to available therapies, confirm resistance to already administered drugs, or explore the activity of new drugs or combinations accessible through clinical trials or compassionate use. Since this was an observational study, patients were finally treated following the clinical decisions of physicians in the most benefit of patients regardless of the results obtained at the PDOs.

Olaparib was tested in all 11 PDOs from epithelial ovarian cancers (Figs. 5, 6 and 7A). Cases #2 and #6 were on or had been previously exposed to PARPi by the time of sample obtention and both patients showed a poor clinical response to the treatment (Figs. 5B and 6C). Accordingly, both showed the lowest sensitivity score to Olaparib (0.5) of the series (Fig. 9A and B). On the contrary, cases #1 #5 and #7 were PARP naïve and showed a higher sensitivity index (Figs. 5A, 6B, 7A and 9B). Tough case #5 did not receive PARPi the patient was deemed platinum sensitive with a platinum free interval longer than 13 months, pointing to a BRCANESS clinical phenotype (Fig. 6B). Finally, sensitivity index to olaparib in case #7 was above one baseline and raised to 3.2 after neoadjuvant chemotherapy. This patient presented a somatic BRCA2 mutation and remained disease free after more than 18 months on adjuvant niraparib (Fig. 7A).

Platinum derivates, carboplatin or cisplatin, were tested in up to 10 of 11 epithelial ovarian cancer PDOs (Fig. 9C). All platinum naïve PDOs were established from samples obtained in the diagnostic laparoscopy of three patients that received carboplatin as neoadjuvant treatment (#3, #4, #7). All three cases presented clinical partial responses and their correspondent PDOs showed a sensitivity score above 1 (Fig. 5C, 6A, 7A and 9B). In cases #4 and #7 metachronous PDOs (established before and after neoadjuvant chemotherapy) showed a rise of the sensitivity score to platinum (from 2.4 to 2.7 in case #4 and from 1.2 to 2.5 in case #7) after treatment exposure. Both source patients were platinum sensitive with platinum free interval (PFI) longer than 6 months.

Synchronous PDOs from cases #6 and #7 showed similar patterns of drug sensitivity regardless of the origin of the sample (tumor tissue or peritoneal wash) (Fig. 9A).

Regarding other histologies, case #8 (Fig. 7B) was a metastasic non-small cell lung cancer harboring a somatic KRAS G12A pathogenic variant. Based on the attending physician recommendations paclitaxel, everolimus and the KRAS inhibitor AMG510 were tested. Concordantly with its mechanism of action, AMG510 presented the lowest sensitivity score since it targets selectively the KRAS G12C mutation and not the KRAS G12A variant of the case studied.

Finally, case #9 (Fig. 7C) was a struma ovarii cancer that had been erroneously diagnosed as a mixed ovarian carcinoma with an endometroid and clear cell components at initial surgery. By the time of first recurrence, proper histological diagnosis was established and a thyroidectomy followed by the administration of I-131 was performed. When disease became Iodium refractory a PDO was established from a metastasis at the liver. Sorafenib presented the highest sensitivity score and was administered to the patient reaching a partial response lasting longer than 10 months.

### PDO establishment from circulating tumor cells (CTCcs)

Peripheral blood samples were obtained from 39 patients (24 [61.5%] females and 15 [38.5%] males), with eleven different histologies (5 endometroid carcinoma, 5 bladder, 4 cervix, 11 ovarian cancer, 2 melanoma, 1 Merkel Cell carcinoma, 4 prostate cancer, 4 renal cancer, 1 adrenal carcinoma, 1 pancreas and 1 uterus carcinoma).

Following the protocol detailed in Supplementary Information, 10 PDOs were successfully established (3 from endometroid cancer patients, 3 from ovarian cancer patients, 2 from renal cancer patients, 1 from a urothelial cancer patient and 1 from a pancreas cancer patient) (Figure S3). However, PDOs did not reach sufficient size or number of cells for further characterization.

### Immuno-organoids

**Fig. 10:**
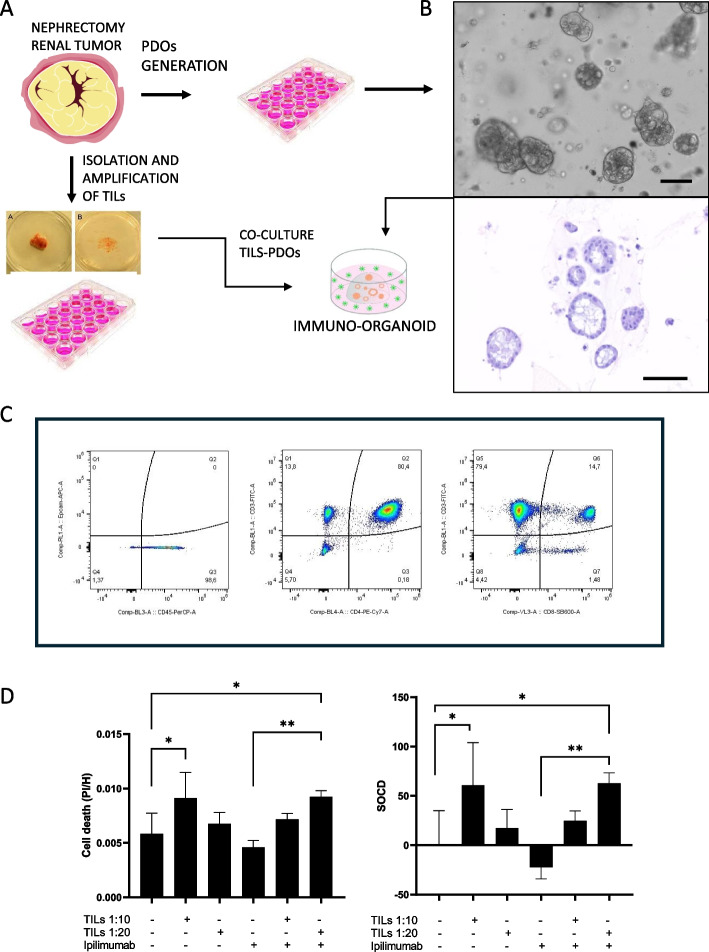
Immunoorganoids establishment and check point inhibitors testing. **A** Schematic representation of the experimental workflow: patient-derived organoids (PDOs) generation, tumor-infiltrating lymphocytes (TILs) isolation and expansion, followed by co-culture of PDOs and TILs to form the immuno-organoid. **B** Bright-field microscopy and hematoxylin–eosin (H&E) staining of generated PDOs, illustrating structural integrity. **C** Phenotypic characterization of expanded TILs using flow cytometry, identifying key immune subpopulations. **D** Cytotoxicity assessment: analysis of cell death ratio (PI/Hoechst) and treatment-specific organoid cell death (SOCD) after co-culturing PDOs with TILs at different ratios (1:10 and 1:20) in the presence or absence of Ipilimumab (38.8 μg/ml). Results are represented as the average of three technical replicates*p < 0.05; **p < 0.0015

Tumor sample of 1 cm^3^ was obtained from a 58-y-old male who underwent a radical nephrectomy because of a 5 cm renal mass. Anatomo-pathological diagnosis corresponded to a type I papillary renal cancer stage I (pT1 N0 M0). Cancer cells were processed as usual while autologous TILs were isolated, characterized and expanded as described in the Materials and Methods section. Once PDOs were established and TILs growth reached at least 2 × 10^6^ cells both were cocultured at three TILs:tumoral cells ratios (1:1, 10:1 and 20:1) for 72 h, in the presence or absence of ipilimumab (anti-CTLA4). No antitumoral activity was seen at the 1:1 ratio (data not shown), but co-cultures with TILs alone resulted in increased organoid cell death in the 10:1 and 20:1 ratios (60,6% and 17,2% respectively). This activity was further enhanced in the ratio 20:1 in the presence of ipilimumab (85,4% vs 17.2% cell death [*p* = 0.0134]. Treatment of PDOs with ipilimumab alone, in the absence of TILs, did not significantly affect organoid viability (Fig. 10).

Source patient did not require adjuvant treatment and remains disease free two years after nephrectomy.

## Discussion

We present the results of a pooled analysis of three prospective multicenter studies that evaluated the viability of cancer Patient-Derived Organoids (PDOs) established from various sources and compared their characteristics and drug sensitivity with the original tumors and the clinical outcome of patients. The establishment success rate of PDOs was 30% (similar from both tumor tissue and peritoneal fluids [39% and 31% respectively]). PDOs preserved the characteristics of the original neoplasias, mimicking patients' treatment responses along the evolution of the disease.

The growing incorporation of new cancer treatments has provided an unprecedented therapeutic arsenal for doctors and patients. Consequently, clinical guidelines often include multiple treatment options for a single indication [34]. While Next Generation Sequencing (NGS) panels can guide therapy selection in some cases, cancer’s complexity extends beyond genetic alterations [35]. Cell plasticity, microenvironment adaptability, and immune response evolution play crucial roles in tumor response to treatment [36, 37].

Therefore, cancer should not be seen merely as a collection of cell clones but as a complex ecosystem where spatial distribution and cell-to-cell interactions are as crucial as genetic variants in determining a patient’s progression [38].

As a result, in recent years, systems aiming to replicate the biological behavior of tumors in vitro have multiplied [14, 15, 39]. Thus, various patient-derived models have been developed to study tumor drug sensitivity and response in an ex vivo setting. These include mouse xenografts, organotypic cultures (also known as tumor slices), and in vitro 2D and 3D cell culture systems [40]. Xenograft models, while biologically relevant, are time-consuming and costly. Organotypic cultures preserve the native tumor architecture and therefore offer a good representation of the tumor microenvironment (TME); however, they require surgical tissue samples, which are not always feasible to obtain.

In vitro models are generally more accessible. Traditional drug testing has relied heavily on 2D cultures due to their simplicity and ease of manipulation. However, in the last two decades, 3D models such as spheroids and organoids have gained prominence, as they better replicate key features of the in vivo tumor environment.

In this regard, patient-derived organoids offer several advantages: they can be derived from small tumor samples in a short time, are cost-effective, and avoid animal use [16]. They represent an accessible, scalable option that may soon be feasible in clinical settings. However, extensive testing in multiple scenarios is necessary before PDOs can become routine in therapeutic decision-making.

Spheroids, while easier to generate and not requiring a supporting matrix, reproduce certain aspects of the in vivo setting—such as oxygen and drug diffusion gradients and cell–cell interactions. However, because they are formed from cell aggregates rather than intact tissue, they lack the structural complexity and heterogeneity characteristic of PDOs, which originate directly from tumor fragments.

In this study, we established PDOs from cancer cases in various clinical settings, not limited to a specific histology with early to heavily pre-treated cases. This broad, heterogeneous approach can provide relevant insight into PDOs’ potential utility in patient care.

One of the first issues to address was the feasibility of establishing PDOs from samples obtained from different hospitals. This is especially relevant when variables such as the time from sample collection to processing or the need for prior freezing have been shown to negatively impact the success rate of PDO establishment [41–43].

In our series, depending on the tumor type, processing the sample immediately (on the same day of extraction) or with a delay (the following day or after freezing) was critical for some tumors (e.g., prostate cancer or ovarian, which presented poorer growth with delayed processing), while it had no effect on others (such as melanoma) Fig. S1. Therefore, establishing PDOs from different tumors requires not only specific culture media but also precise conditions regarding the time from sample extraction to processing. These factors will be essential when incorporating these models into clinical practice, as they will require adapting logistical conditions to the specific type of tumor being studied.

Another point of interest in our work was the possibility of establishing PDOs across a wide range of tumors. There was some variability in the PDO establishment success rate among the most represented histologies (ranging from 33% for ovarian cancer to 68% for renal cancer), which could not be assessed for tumors with few cases.

The success rate in PDO establishment, as previously described [44], varies significantly among different tumor types due to biological and technical factors. There are several reasons that may account for these differences. On one hand, the presence of contaminating epithelial cells can lead to the generation of epithelial organoids, which grow faster than tumor-derived organoids [19]. On the other hand, the composition of culture media used differs between the PDOs of different histological types. The culture conditions used for various organoids are largely based on the seminal work by Sato et al. [45] on benign intestinal organoids. Since then, researchers have empirically optimized media for specific tumor organoids. This lack of standardization in protocols has a direct impact on success rates. Moreover, it has been observed that in certain PDO cultures, specific components of the media can create selective pressures, leading to the expansion of specific subclones, which may result in culture failure or the growth of PDOs that are not representative of the original tumor [44].

The type of matrix used (Matrigel, BME, etc.) can also play a role in the successful of PDOs establishment due to differences in batch-to-batch composition. The development of 3D bio-printing technology together with PDOs, are allowing the development of new cancer models, where vasculature and even nervous and immune components can be added, [46, 47] being more representative of the original tumor and its microenvironment.

This difference in the success rate is undoubtedly one of the limiting steps of any technology based on real patient samples, as the conditions specific to each sample (percentage of tumor content, percentage of necrosis, time since prior cytotoxic treatment administration, etc.) are combined with the biological constraints of each histology (low growth rate of low-grade tumors, requirement for paracrine stimulating factors, etc.) [44].

These differences have critical implications, as they can introduce biases into preclinical research, impact the development of personalized therapies, and limit the representativeness of certain tumor types in precision medicine studies. Therefore, it is essential to consider these variations and to standardize protocols, quality controls, and materials when designing studies and interpreting PDO-based results.

In our series, we were able to confirm that most PDOs, despite originating from samples with different sources and regardless of prior therapies received, retained the biological characteristics of the original tumor and mimicked the clinical behaviour of the patients.

Consistent with the literature, we found that PDOs displayed the same pathogenic variants as the original tumors and showed a similar structural pattern and marker expression by immunohistochemistry [25, 48] (Figs. 3 and 4).

We also evaluated the feasibility of obtaining PDOs from samples of different origins and compared their characteristics both with the original tumor and among organoids from the same patient obtained synchronously or metachronously. Both tumor tissue and peritoneal fluids proved to be efficient sources of viable tumor cells, while extracting Circulating Tumor Cells (CTCs) from peripheral blood resulted in a lower success rate. Additionally, the growth rate of these PDOs was very low, preventing the completion of characterization studies to properly confirm that the organoids obtained were derived from tumor cells.

This finding is consistent with the literature. Numerous factors can influence the success of establishing PDOs from CTCs [49]. The first consideration is that these cells are scarce in the bloodstream, making their isolation and enrichment a crucial step. The biology of CTCs itself reveals that they are heterogeneous populations, not only across different tumor types but also within the same patient [50], and they can be found in the bloodstream as individual cells or as clusters—either homotypic (only CTCs) or heterotypic (CTCs together with other cell types such as immune cells or platelets) [51].

Additionally, CTCs may exhibit various states of epithelial-mesenchymal and mesenchymal-epithelial transition [49], which influence their metastatic potential. Furthermore, the bloodstream represents a "hostile environment" for tumor cells, as they must evade the immune system and withstand fluid shear stress (FSS), which affects cell viability [49, 51, 52].

Together, all these factors impact the isolation of CTCs—whether through physical or biological methods—a vital step for the establishment of PDOs.

Moreover, several studies have shown that the production of CTCs by breast tumors and their metastatic potential follow a circadian rhythm [53–55]. Therefore, the time at which the cells are extracted from the patient could also affect their subsequent proliferative capacity, hindering in vitro growth.

Once CTCs are isolated and enriched, in vitro growth presents additional challenges such as interactions with culture matrices (not all matrices are suitable for all cell types); selection of the appropriate culture media (as each CTC type may require specific growth factors, hormones, or other agents); and oxygen levels (generally, CTCs grow better under hypoxic conditions, although this varies among CTCs) [49].

Lastly, some researchers have observed a degree of differentiation in PDOs derived from CTCs after several passages, highlighting the need to periodically assess their genetic and phenotypic characteristics [56].

Thus, given the difficulty in obtaining PDOs from peripheral blood, the ability to establish ascitic fluid as a suitable source of viable cells is particularly important [22, 23]. Many tumors typically exhibit a pattern of peritoneal dissemination, leading to the accumulation of ascitic fluid, which is relatively accessible for serial sampling. In this way, as we were able to do in some cases in our series, we could establish PDOs at different points throughout the disease, adapting therapeutic decisions based on the most recent sensitivity patterns. This possibility opens a completely new scenario for PDO use, more versatile and dynamic than the traditional drug testing on a single isolated sample collected at a specific point in time.

In this regard, cases #4 and #7 are particularly representative, as PDOs were obtained both before and after platinum exposure (Figs. 6A, 7A). In both cases, treatment sensitivity increased in the second sample, consistent with the evolution of the"source"patients, who proved to be platinum sensitive.

Similarly, cases #2 and #6, (Figs. 5B, 6C) whose PDOs were established after exposure to PARPi and showed a low sensitivity score to olaparib, experienced rapid progression on these treatments.

Another interesting aspect was the inability to establish absolute thresholds for sensitivity and resistance. Although PDOs sensitive to platinum and PARPis showed sensitivity scores above 1, some values were very close to this threshold. Additionally, as mentioned earlier, the score value varied over time in serial cases. Finally, in resistant patients, although their scores were below 1, the values were also close to this cut-off point. Therefore, it seems that we cannot define a fixed threshold to guarantee a patient's response to a treatment; instead, we should establish a relative order of sensitivity for the drugs tested in each case.

Thus, when predicting a tumor's sensitivity to different drugs, it may be essential to know the history of prior therapy exposure, including, if possible, sensitivity values from previous tests.

These results provide a new approach to using PDOs, where close contact with the patient's physicians can be crucial not only in determining which drugs should be studied but also in interpreting the results. Therefore, collaboration between clinicians and basic scientists will be essential to fully leverage the potential of a tool like PDOs, which, when integrated into the proper clinical context, could provide complementary information to other molecular techniques like NGS.

Following this same approach and given that this study involved a heterogeneous cohort not limited to a single tumor type or predefined clinical situation, we opted to collaborate with each patient’s physician to determine the drugs to be tested in vitro. Drugs were selected based on clinical criteria, prioritizing information on the sensitivity profile to available therapies or evaluating potentially accessible alternatives through clinical trials or compassionate use programs. One particularly interesting case involved an ultra-rare tumor (struma ovarii) [57], allowing us to test different treatment options for a disease with no approved drugs or specific clinical trials (Fig. 7C). Although therapeutic decisions were made independently of the study results, the fact that the drug with the highest in vitro activity was sorafenib enabled the treating physician to confirm their initial choice, supporting the authorization for compassionate use of the drug.

Another notable aspect of this case is that sorafenib demonstrated greater in vitro efficacy than lenvatinib. Although both drugs have the ability to inhibit RET—a common driver gene in thyroid cancer [58, 59]—lenvatinib is considered more potent. Unfortunately, lenvatinib is associated with higher toxicity and significantly greater cost, factors that are especially relevant when considering off-label treatments [60, 61]. The PDO results correctly identified sorafenib as the preferred option, leading to a radiological response in the patient that persisted for 10 months, with no appreciable toxicity. Cases like this highlight a new scenario for the use of PDOs.

It is well established that rare tumors collectively account for approximately 20% of cancer diagnoses and generally have a worse prognosis than more common pathologies. This prognosis is partly due to the lack of access to novel medications, as most therapies do not have development programs for these indications [62]. The ability to study multiple pharmacological options in patient-derived organoids (PDOs) from individuals with rare tumors could help address this inequity by identifying therapeutic options useful for each unique case.

Equally innovative was the ability to test an antibody–drug conjugate (ADC), enfortumab, vedotin in one of our models. (Fig. 7A) This drug targets the nectin-4 protein, which is overexpressed in various tumors, including ovarian cancer [63–65]. Although, in our case, no in vitro activity was observed and the patient did not receive the drug, it opens a new possibility for using PDOs in cancer research. It is well established that many ADCs exhibit a bystander effect, where cells near the initial target cell are damaged by proximity when the payload (cytotoxic drug linked to the ADC) is released [66]. This effect can be significant, allowing some patients to experience substantial responses to specific ADCs even with very low expression of the target protein. PDOs, with their three-dimensional configuration, could more accurately model this therapeutic effect, facilitating their development and better identifying responsive cases compared to simple immunohistochemical staining.

Finally, and also of great interest, is the potential to develop co-cultures of patient-derived organoids (PDOs) and tumor-infiltrating lymphocytes (TILs) to predict sensitivity to immunotherapy [65–67]. In our case, the antitumor activity of lymphocytes cultured in vitro, as well as the effect of drugs such as the checkpoint inhibitor ipilimumab, was demonstrated (Fig. 10). If consolidated, this technique could represent a significant advancement in the development of immunotherapy in the coming years.

Unfortunately, our study is not without limitations. Although it is not restricted to a specific tumor type, there is an enrichment of certain neoplasms (such as ovarian or prostate cancer) at the expense of others (such as lung or breast cancer) due to the clinical practices of collaborating physicians specializing in specific oncology fields. This could limit our ability to generalize the results to other neoplasms.

Another significant bias, inherent in any patient study, is the accessibility to suitable samples and the biases related to some individuals’ predisposition to participate in research studies. Additionally, ethnic representation was very limited, as all cases except one of Asian origin were Caucasian. Nevertheless, the multicenter nature of the study may partially mitigate this limitation, as it allowed the inclusion of a broad and heterogeneous patient variety.

In summary, PDOs from cancer patients retain the morphological and genetic characteristics of the original cases, reflecting patients' real clinical evolution through their in vitro drug sensitivity. PDOs can be established from both tumor tissue and ascitic fluid, showing very similar behaviour. This is especially important because it opens the door to less invasive and more comfortable sampling methods for patients, making it possible to collect follow-up samples over time and monitor tumor progression in the laboratory. The ability to obtain serial PDOs allows for the dynamic observation of drug sensitivity patterns over time, providing potentially valuable information for clinicians in selecting the best therapy among routine options or drugs in development or accessible through compassionate use. Direct interaction with the physician responsible for each case can be crucial, not only for selecting the most relevant drugs for study but also for understanding prior drug exposure, each patient’s genetic background, and even for interpreting the results. This approach ensures that the information provided by the models will be significantly more useful.

Instead of attempting to establish fixed thresholds that alone predict whether a case will respond to a specific treatment, as has been done previously, it is likely that the information provided by PDOs should be interpreted relatively. That is, they will allow for the comparison of different options among themselves and indicate which may be more effective. However, the practical application of this information will require a multidisciplinary environment with the direct involvement of the physicians responsible for each case.

Of course, bringing this technology into routine clinical use will require further refinement and standardization of organoid culture protocols. This might include adding other cell types and elements of the tumor microenvironment—like immune cells, fibroblasts, or endothelial cells—to better reflect the complexity of real tumors. Newer approaches, such as bioprinting and microfluidic systems, could also help improve consistency, scalability, and automation of the process.

In recent years, more and more clinical trials have started using PDOs to predict how patients might respond to treatment [68]. The results from these studies will be key to understanding just how far this technology can go in helping make personalized medicine a reality.

## Conclusions

Our findings demonstrate that PDOs established from different sources preserve tumor characteristics, reflect disease progression, and can predict treatment response, making them ideal models to complement molecular testing in precision medicine. Our study also highlights the strong potential of PDOs to identify optimal therapeutic strategies in rare tumors, which often lack specific treatment programs. The significance of this work lies in demonstrating the ability of PDOs to enhance therapy selection, enable dynamic monitoring of drug response, support research into rare cancers and complex drug mechanisms, and ultimately help realize the promise of personalized medicine when integrated with clinical data and interpreted through multidisciplinary collaboration.

## Supplementary Information

Supplementary Material 1.

## Data Availability

No datasets were generated or analysed during the current study.

## Abbreviations

2D: Two Dimensional
3D: Three dimensional
ADCs: Antibody-drug conjugates
AUC: Area Under the Curve
Cmax: Maximun plasma concentration
CTCs: Circulating Tumoral Cells
DAB-30: Diaminobenzidine tetrahydrochloride
DMSO: Dimethyl sulfoxide
FBS: Fetal Bovine Serum
H&E: Hematoxylin and Eosin
IHC: Immunohistochemistry
NGS: Next Generation Sequencing
PBS: Phosphate-Buffered Saline
PCR: Polymerase Chain Reaction
PDO: Patient-Derived Organoids
PDTX: Patient-derived Tumor Xenograft
PFI: Platinum Free Interval
PI: Propidium Iodide
SOCD: Treatment-Specific Organoid Cell Death
STS: Staurosporine
TCM: T Central Memory Cell
Tc: T.Cytotoxic Cell
TEMRA: Terminal Effector T Cells
TEM: T Effector Memory Cell
Th: T Helper Cell
TILs: Tumor-Infiltrating Lymphocytes
TN: Naïve T Cells
Tregs: T Regulatory Cells
UKN: Uknown
WHO: World Health Organization
