# Supplementary material for: Multicenter study correlating molecular characteristics and clinical outcomes of cancer cases with patient-derived organoids
Source: J Exp Clin Cancer Res. 2025 Jul 2;44:182. doi: 10.1186/s13046-025-03437-0 (PMC12220348; doi:10.1186/s13046-025-03437-0)
Supplement: Supplementary file 1 — Supplementary Material 1. [file 13046_2025_3437_MOESM1_ESM.docx]

**SUPPLEMENTARY MATERIAL**

**SUPLEMENTARY FIGURES**


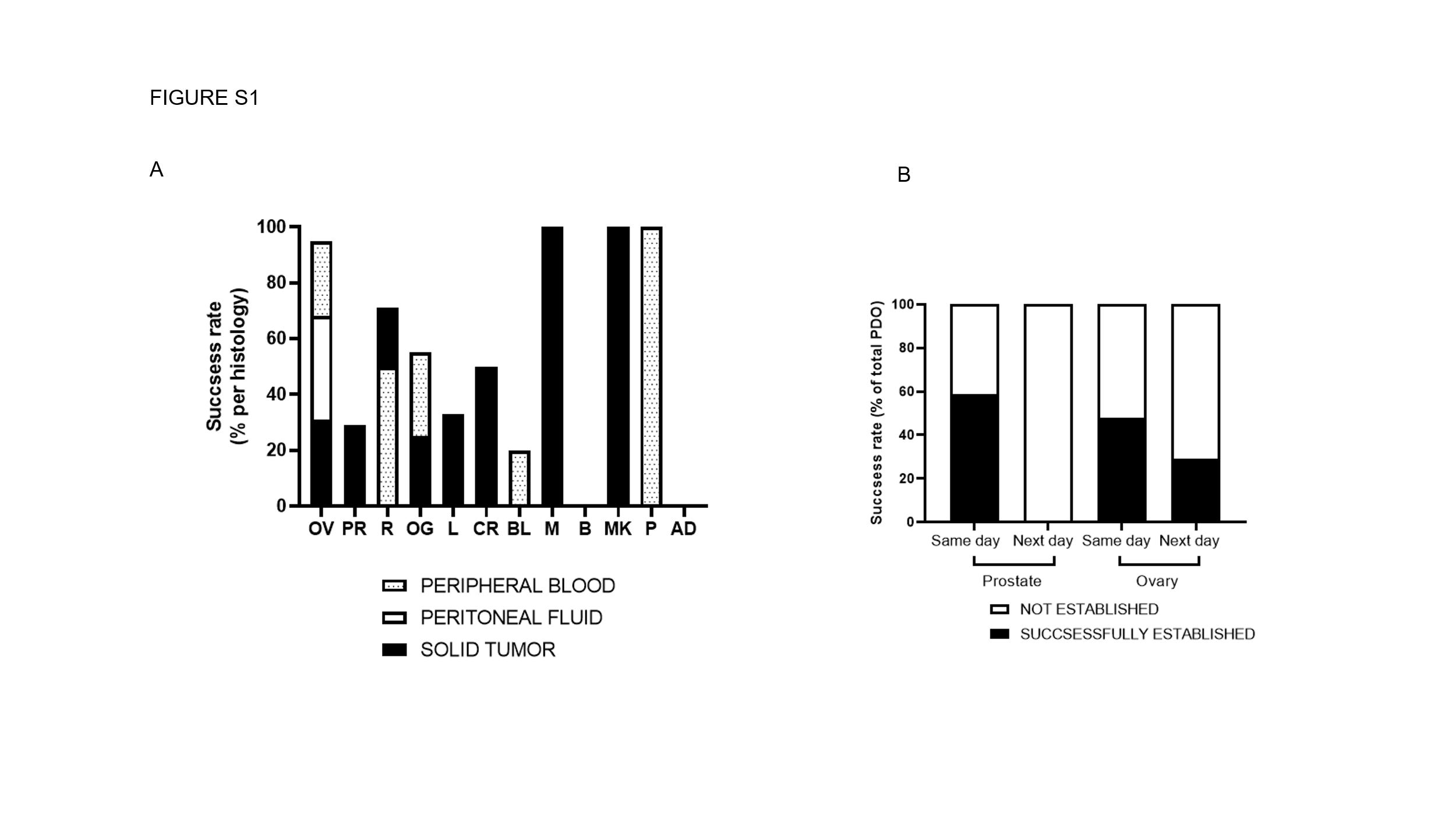


**Figure S1: Success rate in PDOs establishment.** (A) Success rate measured as percentage of PDOs established respect to the total of samples per type of tumor. (B) Success rate with ovary and prostate samples processed the same day of surgery/biopsy or the following day. OV, ovary; PR, prostate; R, renal; OG, other gynecological; L, lung; CR, colorectal; BL, bladder; M, melanoma; B, basocellular; MK, merkel; P, pancreas; AD, adrenal


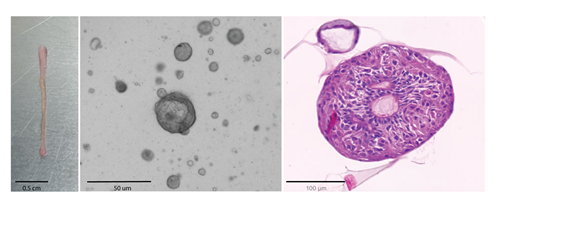


**A**

**B**

**C**

**Figure S2: Prostate Organoids morphology.** (A) Fragment of biopsy. (B) Representative image of a prostate PDO in a bright field. Scale bar=50um. (B) Haematoxilyn and Eosin staining of PDO. Scale bar=100um

**
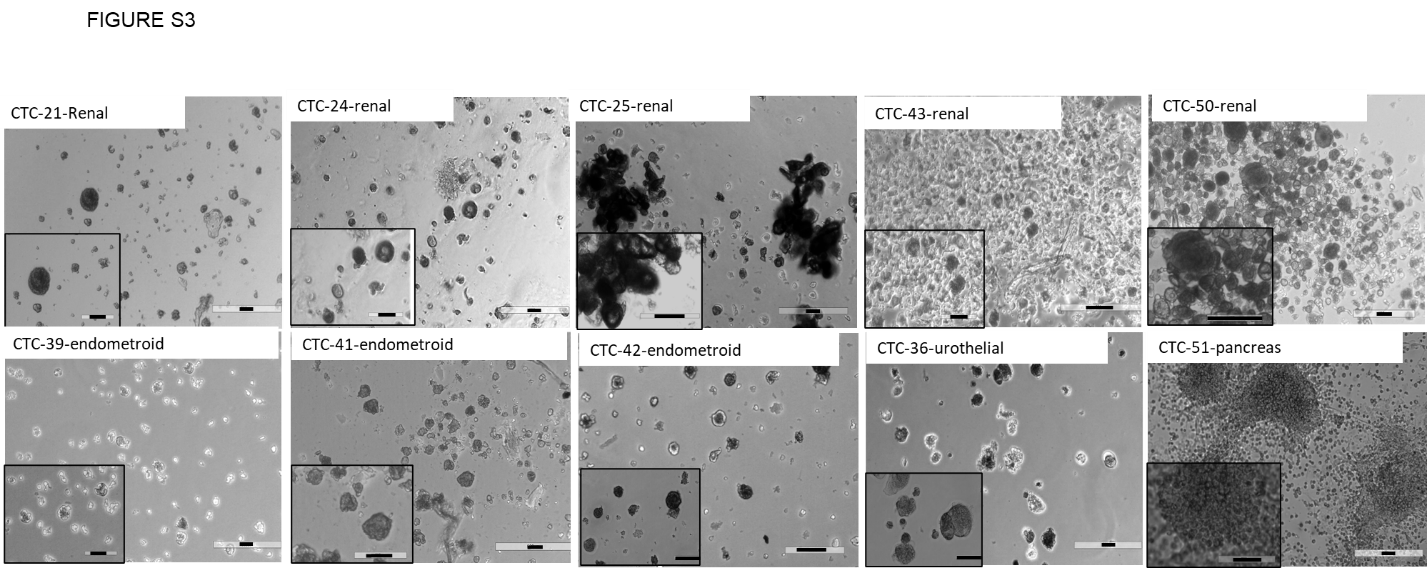
**

**Figure S3: PDOs derived from circulating tumoral cells (CTCs).** Representative images of PDOs generated from CTCs isolated from peripheral blood. The size of the black scale bar 100um.

**Table S3: Success rates per histology**

| **TYPE OF TUMOR** | **SAMPLES (N)** | **ORGANOIDS ESTABLISHED (N)** | **SUCCSESS RATE (%)** |
| --- | --- | --- | --- |
|  |  |  |  |
| **OVARY** | 139 | 46 | 33 |
| **PERITONEAL FLUID** | 57 | 21 | 37 |
| **SOLID TUMOR** | 71 | 22 | 31 |
| **PERIPHERAL BLOOD** | 11 | 3 | 27 |
| **PROSTATE** | 34 | 10 | 29 |
| **SOLID TUMOR** | 30 | 10 | 33 |
| **PERIPHERAL BLOOD** | 4 | 0 | 0 |
| **RENAL** | 28 | 19 | 68 |
| **SOLID TUMOR** | 24 | 17 | 71 |
| **PERIPHERAL BLOOD** | 4 | 2 | 50 |
| **OTHER GYNECOLOGYC** | 21 | 5 | 24 |
| **PERITONEAL FLUID** | 3 | 0 | 0 |
| **SOLID TUMOR** | 8 | 2 | 25 |
| **PERIPHERAL BLOOD** | 10 | 3 | 30 |
| **LUNG** | 7 | 2 | 29 |
| **PERITONEAL FLUID** | 1 | 0 | 0 |
| **SOLID TUMOR** | 6 | 2 | 33 |
| **COLORECTAL** | 4 | 2 | 50 |
| **SOLID TUMOR** | 4 | 2 | 50 |
| **BLADDER** | 5 | 1 | 20 |
| **PERIPHERAL BLOOD** | 5 | 1 | 20 |
| **MELANOMA** | 5 | 3 | 60 |
| **SOLID TUMOR** | 3 | 3 | 100 |
| **PERIPHERAL BLOOD** | 2 | 0 | 0 |
| **BASOCELULAR** | 1 | 0 | 0 |
| **SOLID TUMOR** | 1 | 0 | 0 |
| **MERKEL** | 2 | 1 | 50 |
| **SOLID TUMOR** | 1 | 1 | 100 |
| **PERIPHERAL BLOOD** | 1 | 0 | 0 |
| **PANCREAS** | 2 | 1 | 50 |
| **SOLID TUMOR** | 1 | 0 | 0 |
| **PERIPHERAL BLOOD** | 1 | 1 | 100 |
| **ADRENAL** | 1 | 0 | 0 |
| Total | **249** | **90** | **36** |

**Table S4: Immunoprofiling (characterization through flow cytometry of the immune infiltrate of the source tumor)**

**Table S5: Sequences of primers used for genetic analysis**

**Table S6: List of drugs and its Cmax concentrations**

**Table S7: Specific media for PDOs culture**

| COLORECTAL CANCER ORGANOIDS MEDIUM | **stock concentration** | **final concentration** |
| --- | --- | --- |
| DMEM F12 /Adcanced medium |  |  |
| Penicillin/Streptomicin | 100x | 1x |
| GLUTAMAX | 100x | 1x |
| HEPES | 1M | 10mM |
| R-Spondin | 500 ug/ml | 100 ng/ml |
| Noggin | 100 ug/ml | 50 ng/ml |
| EGF | 500 ug/ml | 50 ng/ml |
| B27 | 50x | 1x |
| Nicotinamide | 100x 1M | 10 mM |
| N-Acetilcysteine | 400x 500mM | 1,25 mM |
| SB202190 | 30mM | 3 uM |
| A8301 | 50000x 25mM | 500 nM |
| Y27632 | 100mM | 10 uM |
| GASTRIN I | 0,5mg/ml | 50ng/ml |
|  |  |  |
| PANCREAS CANCER ORGANOIDS MEDIUM | **stock concentration** | **final concentration** |
| DMEM F12 /Adcanced medium |  |  |
| Penicillin/Streptomicin | 100x | 1x |
| GLUTAMAX | 100x | 1x |
| HEPES | 1M | 10mM |
| R-Spondin | 500 ug/ml | 100 ng/ml |
| Noggin | 100 ug/ml | 100 ng/ml |
| EGF | 500 ug/ml | 50 ng/ml |
| B27 | 50x | 1x |
| Nicotinamide | 100x 1M | 10 mM |
| N-Acetilcysteine | 400x 500mM | 1 mM |
| FGF10 | 100ug/ml | 100ng/ml |
| A8301 | 50000x 25mM | 500 nM |
| Primocin | 50mg/ml | 1mg/ml |
| GASTRIN I | 0,5mg/ml | 50ng/ml |
|  |  |  |
| OVARIAN CANCER ORGANOIDS MEDIUM | **stock concentration** | **final concentration** |
| Penicillin/Streptomicin | 100x | 1x |
| GLUTAMAX | 100x | 1x |
| HEPES | 1M | 10mM |
| R-Spondin | 500 ug/ml | 100 ng/ml |
| Noggin | 100 ug/ml | 100 ng/ml |
| EGF | 500 ug/ml | 50 ng/ml |
| B27 | 50x | 1x |
| Nicotinamide | 100x 1M | 5 mM |
| N-Acetilcysteine | 400x 500mM | 1,25 mM |
| SB202190 | 3000x 30mM | 1x, 10 uM |
| A8301 | 50000x 25mM | 1,25uM |
| Y27632 | 100mM | 10 uM |
| HGF | 100ug/ml | 10ng/ml |
| Heregulina | 0.1mg/ml | 50ug/ml |
| IGF-1 | 0.1mg/ml | 20ng/ml |
| B-Estradiol | 100uM | 10nM |
|  |  |  |
| LUNG CANCER ORGANOIDS MEDIUM | **stock concentration** | **final concentration** |
| Penicillin/Streptomicin | 100x | 1x |
| GLUTAMAX | 100x | 1x |
| HEPES | 1M | 10mM |
| EGF | 500 ug/ml | 50 ng/ml |
| FGF2 | 50 ug/ml | 20 ng/ml |
| B27 | 50x | 1x |
| Y27632 | 100mM | 10 uM |
|  |  |  |
| PROSTATA CANCER ORGANOIDS MEDIUM | **stock concentration** | **final concentration** |
| Penicillin/Streptomicin | 100x | 1x |
| GLUTAMAX | 100x | 1x |
| HEPES | 1M | 10mM |
| R-Spondin | 500 ug/ml | 500 ng/ml |
| Noggin | 100 ug/ml | 100 ng/ml |
| EGF | 500 ug/ml | 5 ng/ml |
| B27 | 50x | 1x |
| Nicotinamide | 100x 1M | 10 mM |
| N-Acetilcysteine | 400x 500mM | 1,25 mM |
| SB202190 | 3000x 30mM | 1x, 10 uM |
| A8301 | 50000x 25mM | 2,5uM |
| FGF10 | 100ug/ml | 10ng/ml |
| FGF2 | 50ug/ml | 5ng/ml |
| Prostaglandin E2 | 10000x 10mM | 1 uM |
| Y27632 | 100mM | 10 uM |
| DHT | 1uM | 1nM |
|  |  |  |
| KIDNEY CANCER ORGANOIDS MEDIUM | **stock concentration** | **final concentration** |
| DMEM F12 /Adcanced medium |  |  |
| Penicillin/Streptomicin | 100x | 1x |
| GLUTAMAX | 100x | 1x |
| HEPES | 1M | 10mM |
| EGF | 500 ug/ml | 20 ng/ml |
| B27 | 50x | 1x |
| FGF10 | 100ug/ml | 20ng/ml |
| Y27632 | 100mM | 10 uM |
|  |  |  |
| BLADDER CANCER ORGANOID MEDIUM | **stock concentration** | **final concentration** |
| DMEM F12 /Adcanced medium |  |  |
| Penicillin/Streptomicin | 100x | 1x |
| GLUTAMAX | 100x | 1x |
| HEPES | 1M | 10mM |
| R-Spondin | 500 ug/ml | 100 ng/ml |
| Noggin | 100 ug/ml | 100 ng/ml |
| A8301 | 25mM | 5uM |
| N-Acetilcysteine | 400x 500mM | 1,25 mM |
| Nicotinamide | 100x 1M | 10 mM |
| EGF | 500 ug/ml | 20 ng/ml |
| B27 | 50x | 1x |
| FGF10 | 100ug/ml | 10ng/ml |
| FGF2 | 50ug/ml | 12,5ng/ml |
| SB202190 | 30 mM | 10 uM |
| Heregulina | 0.1mg/ml | 50ug/ml |
| Y27632 | 100mM | 10 uM |
|  |  |  |
|  |  |  |
| MELANOMA ORGANOID MEDIUM | **stock concentration** | **final concentration** |
| DMEM F12 /Adcanced medium |  |  |
| Penicillin/Streptomicin | 100x | 1x |
| GLUTAMAX | 100x | 1x |
| HEPES | 1M | 10mM |
| R-Spondin | 500 ug/ml | 100 ng/ml |
| Noggin | 100 ug/ml | 100 ng/ml |
| A8301 | 25mM | 1,25uM |
| N-Acetilcysteine | 400x 500mM | 1,25 mM |
| Nicotinamide | 100x 1M | 10 mM |
| EGF | 500 ug/ml | 50 ng/ml |
| B27 | 50x | 1x |
| FGF2 | 50ug/ml | 10ng/ml |
| SB202190 | 30 mM | 10 uM |
| Heregulina | 0.1mg/ml | 50ug/ml |
| Y27632 | 100mM | 10 uM |
| Prostaglandina E2 | 10000x 10mM | 1uM |
| Hidrocortisona | 2mM | 10mM |
